# Supplementary material for: Coevolution of religious and political authority in Austronesian societies
Source: Nat Hum Behav. 2022 Nov 10;7(1):38–45. doi: 10.1038/s41562-022-01471-y (PMC9883158; doi:10.1038/s41562-022-01471-y)
Supplement: Supplementary file 1 — Supplementary methods, table and references. [file 41562_2022_1471_MOESM1_ESM.pdf]

nature human behaviour

Article

https://doi.org/10.1038/s41562-022-01471-y

Coevolution of religious and political authority in Austronesian societies

In the format provided by the authors and unedited

Supplementary Methods

Static phylogenetic generalised linear mixed model

Our static Bayesian phylogenetic generalised linear mixed model has the form:

$$\text{Authority}_{[i,j]} \sim \text{OrderedLogistic}(\eta_{[i,j]}, c_{[j]})$$

$$\eta_{[i,j]} = \alpha_{[j]} + \text{phy}_{[i,j]} + \text{res}_{[i,j]}$$

$$\text{phy}_{[i,j]} \sim \text{MVNormal}(\begin{bmatrix} 0 \\ \ldots \\ 0 \end{bmatrix}, \Sigma_{\text{phy}})$$

$$\text{res}_{[i,j]} \sim \text{MVNormal}(\begin{bmatrix} 0 \\ \ldots \\ 0 \end{bmatrix}, \Sigma_{\text{res}})$$

$$\Sigma_{\text{phy}[j]} = \begin{bmatrix} \sigma_{\text{phy}[1]} & 0 \\ 0 & \sigma_{\text{phy}[2]} \end{bmatrix} \Omega_{\text{phy}} \begin{bmatrix} \sigma_{\text{phy}[1]} & 0 \\ 0 & \sigma_{\text{phy}[2]} \end{bmatrix}$$

$$\Sigma_{\text{res}[j]} = \begin{bmatrix} \sigma_{\text{res}[1]} & 0 \\ 0 & \sigma_{\text{res}[2]} \end{bmatrix} \Omega_{\text{res}} \begin{bmatrix} \sigma_{\text{res}[1]} & 0 \\ 0 & \sigma_{\text{res}[2]} \end{bmatrix}$$

$$\text{Phylogenetic Signal}_j = \frac{\text{var}(\text{phy}_{[j]})}{\text{var}(\eta_{[j]}) + \frac{\pi^2}{3}}$$

where *phy* and *res* are random effects that capture the phylogenetic variance-covariance and the residual variance-covariance, respectively. $\frac{\pi^2}{3}$ is the latent scale variance induced by the logit link function[1]. The phylogenetic correlation is given by the off-diagonals of the Ω_{phy} matrix. We fitted this model with the *brms* R package[2] using weakly regularising priors. To account for phylogenetic uncertainty, we combined the posteriors across 100 models fitted to 100 randomly-drawn posterior phylogenetic trees. Standard MCMC diagnostics ($\hat{R} \leq 1.05$) and trace plots suggested that the model converged normally.

Dynamic co-evolutionary model

Adapting previous work[3], we model the coevolution of political and religious authority as a multivariate stochastic differential equation, similar to a multivariate Ornstein-Uhlenbeck (OU) process. OU processes are mean-reverting stationary Gauss-Markov processes, whereby a trait changes due to both deterministic reversion towards some central value and stochastic Gaussian noise. In an evolutionary context, these deterministic and stochastic components of the OU process are often referred to as 'selection' and 'drift', respectively.

Using an OU process, we model the evolutionary history of political and religious authority on the Austronesian language phylogeny as a time series. We allow the deterministic dynamics of the OU process ('selection') to play out over the length of each tree segment, and add the stochastic drift components ('drift') to the end of each segment as independent samples from a standard normal distribution. The differential equation is as follows:

$$d\eta(t) = (\mathbf{A}\eta(t) \ + \mathbf{b})dt + \mathbf{G}dW(t)$$

where $\eta(t)$ is a vector of latent variables, underlying our observed ordinal authority variables, at time t. The matrix $\mathbf{A}$ represents 'selection', with diagonal terms representing autoregressive effects (i.e. the effect of a latent variable on itself) and the off-diagonal terms representing the effect of each latent variable on the other (e.g. if η_1 is political authority and η_2 is religious authority, then $\mathbf{A}[2,1]$ represents the effect of political authority on religious authority). The vector $\mathbf{b}$ is a vector of continuous time intercepts. The matrix $\mathbf{G}$ is the Cholesky decomposition of the 'drift' covariance matrix $\mathbf{Q}=\mathbf{G}\mathbf{G}^{\mathrm{T}}$, which scales the stochastic component of the model.

As outlined in Driver et al.[4] and Ringen et al.[3], the solution to this differential equation for any time interval $t - t_0$ is:

$$\eta(t) = e^{\mathbf{A}(t - t_0)}\eta(t_0) + \mathbf{A}^{-1}\left[e^{\mathbf{A}(t - t_0)}\right]\mathbf{b} + \int_{t_0}^{t} e^{\mathbf{A}(t-s)}\mathbf{G}dW(s)$$

$$\text{cov}\left[\int_{t_0}^{t} e^{\mathbf{A}(t-s)}\mathbf{G}dW(s)\right] = \text{irow}\left(\mathbf{A}_{\#}^{-1}\left[e^{\mathbf{A}_{\#}(\boldsymbol{t}-\boldsymbol{t_0})} - \mathbf{I}\right]\text{row}(\mathbf{Q})\right)$$

where $\mathbf{A}_{\#} = \mathbf{A} \otimes \mathbf{I} + \mathbf{I} \otimes \mathbf{A}$ with$\otimes$ denoting the Kronecker-product, $\mathbf{I}$ is an identity matrix, $\text{row}()$ is an operation that takes elements of a matrix row-wise and puts them in a column vector, and $\text{irow}()$ is the inverse of the row operation.

We map this model onto the Austronesian language phylogeny with the *rstan* R package[5]. Following the algorithm in Ringen et al[3]., we divide the evolutionary history of each lineage into tree segments, where each tree segment begins with the parent node and ends with the child node or tip, and calculate the length of each segment s. We then initialise the ancestral trait values for political and religious authority and, for each segment, solve the above equation for $\eta(s)$. We repeat the above steps for all taxa on the phylogeny. To account for phylogenetic uncertainty, we combined the posteriors across 100 models fitted to 100 randomly-drawn posterior phylogenetic trees. To account for spatial autocorrelation in the locations of Austronesians societies, we include a Gaussian process over longitude and latitude coordinates. Standard MCMC diagnostics ($\hat{R} \leq 1.05$) and trace plots suggested that the model converged normally.

In our dynamic co-evolutionary model with two latent variables, political authority η_1 and religious authority η_2, we can calculate the equilibrium trait values θ for both latent variables as:

$$\theta_{\eta_1} = \frac{-(\mathbf{A}[1,2]\eta_2 + \mathbf{b}_1)}{\mathbf{A}[1,1]}$$

$$\theta_{\eta_2} = \frac{-(\mathbf{A}[2,1]\eta_1 + \mathbf{b}_2)}{\mathbf{A}[2,2]}$$

In the main text, we report the standardised difference in the equilibrium value for one trait, given an absolute deviation increase in the other trait (θ_z).

For further details about this dynamic co-evolutionary model, see Ringen et al.[3] and our R and Stan code at https://github.com/ScottClaessens/phyloAuthority

Supplementary Table: Summary of Multistate Analyses

**Full Model**													
**Run**	**Log Marginal Likelihood**	**Transition Rates**											
		**q01**	**q02**	**q03**	**q10**	**q12**	**q13**	**q20**	**q21**	**q23**	**q30**	**q31**	**q32**
**1**	-131.60	3.09	3.40	2.48	3.09	4.33	3.04	3.40	4.35	4.33	2.72	3.35	4.36
**2**	-131.55	3.02	3.42	2.57	3.09	4.29	3.00	3.38	4.33	4.42	2.77	3.34	4.46
**3**	-131.56	3.04	3.58	2.49	3.11	4.33	2.97	3.56	4.36	4.39	2.67	3.30	4.46
**Mean**	-131.57	3.05	3.47	2.51	3.10	4.32	3.00	3.45	4.35	4.38	2.72	3.33	4.42
**Differentiation Model (Strong Version) (q02 q03 q13=0)**													
**Run**	**Log Marginal Likelihood**	**Transition Rates**											
		**q01**	**q02**	**q03**	**q10**	**q12**	**q13**	**q20**	**q21**	**q23**	**q30**	**q31**	**q32**
**1**	-131.76	7.46	0	0	7.14	7.49	0	0.06	7.30	7.47	0.50	1.61	7.22
**2**	-131.79	7.44	0	0	7.13	7.47	0	0.06	7.28	7.47	0.50	1.58	7.21

**3**	-131.71	7.47	0	0	7.14	7.49	0	0.06	7.29	7.49	0.52	1.62	7.24
**Mean**	-131.75	7.46	0	0	7.14	7.48	0	0.06	7.29	7.48	0.51	1.61	7.22
**Differentiation Model (Weak Version) (q02 q03=0)**													
**Run**	**Log Marginal Likelihood**	**Transition Rates**											
		**q01**	**q02**	**q03**	**q10**	**q12**	**q13**	**q20**	**q21**	**q23**	**q30**	**q31**	**q32**
**1**	-131.89	7.45	0	0	5.20	6.83	4.99	0.10	6.61	6.57	2.37	3.65	6.36
**2**	-131.87	7.36	0	0	5.13	6.71	4.85	0.09	6.48	6.55	2.36	3.58	6.33
**3**	-131.87	7.44	0	0	5.17	6.72	5.08	0.10	6.49	6.43	2.39	3.74	6.22
**Mean**	-131.88	7.42	0	0	5.17	6.75	4.98	0.10	6.53	6.52	2.37	3.66	6.31
**Unification Model (Strong Version) (q01 q02 q31=0)**													
**Run**	**Log Marginal Likelihood**	**Transition Rates**											
		**q01**	**q02**	**q03**	**q10**	**q12**	**q13**	**q20**	**q21**	**q23**	**q30**	**q31**	**q32**
**1**	-132.28	0	0	7.69	0.49	7.57	0.84	0.01	7.72	7.52	7.24	0	7.74

**2**	-132.19	0	0	7.75	0.54	7.59	0.90	0.01	7.78	7.56	7.27	0	7.79
**3**	-132.31	0	0	7.79	0.52	7.67	0.85	0.01	7.82	7.60	7.33	0	7.82
**Mean**	-132.26	0	0	7.75	0.52	7.61	0.86	0.01	7.77	7.56	7.28	0	7.78
**Unification Model (Weak Version) (q01 q02=0)**													
**Run**	**Log Marginal Likelihood**	**Transition Rates**											
		**q01**	**q02**	**q03**	**q10**	**q12**	**q13**	**q20**	**q21**	**q23**	**q30**	**q31**	**q32**
**1**	-132.07	0	0	7.55	2.90	5.90	3.56	0.04	6.03	6.88	4.64	5.71	7.10
**2**	-132.09	0	0	7.64	3.03	6.29	3.54	0.03	6.41	6.81	4.63	5.77	7.01
**3**	-132.13	0	0	7.35	2.82	5.99	3.44	0.03	6.11	6.75	4.55	5.56	6.95
**Mean**	-132.10	0	0	7.51	2.92	6.06	3.51	0.03	6.19	6.81	4.61	5.68	7.02

Supplementary References

1. Nakagawa, S., &Schielzeth, H.A general and simple method for obtaining R^2 from generalized linear mixed-effects model. *Methods Ecol. Evol.***4**, 133-142 (2013).

2. Bürkner, P. *brms*: An R package for Bayesian multilevel models using *Stan*. *J. Stat. Softw.* **80**, 1–28 (2017).

3. Ringen, E. J., Martin, J.S., &Jaeggi, A.V. Novel phylogenetic methods reveal that resource-use intensification drives the evolution of 'complex' societies. Preprint at *EcoEvoR*xiv, https://ecoevorxiv.org/wfp95/ (2021).

4. Driver, C.C., Oud, J. H., &Voelke, C. C. Continuous time structural equation modeling with r package *ctsem. J. Stat. Softw.* **77** (2017).

5. *Stan* Development Team. *RStan: The R interface to Stan (R package version 2.19.3.).*http://mc-stan.org/ (2020).
