# Supplementary material for: Coevolution of religious and political authority in Austronesian societies
Source: Nat Hum Behav. 2022 Nov 10;7(1):38–45. doi: 10.1038/s41562-022-01471-y (PMC9883158; doi:10.1038/s41562-022-01471-y)
Supplement: Supplementary file 2 — Reporting Summary [file 41562_2022_1471_MOESM2_ESM.pdf]

nature portfolio

Corresponding author(s): Oliver Sheehan

Last updated by author(s): March 4th 2022

Reporting Summary

Nature Portfolio wishes to improve the reproducibility of the work that we publish. This form provides structure for consistency and transparency in reporting. For further information on Nature Portfolio policies, see our Editorial Policies and the Editorial Policy Checklist.

Statistics

For all statistical analyses, confirm that the following items are present in the figure legend, table legend, main text, or Methods section.

n/a	Confirmed	
☐	☒	The exact sample size (*n*) for each experimental group/condition, given as a discrete number and unit of measurement
☒	☐	A statement on whether measurements were taken from distinct samples or whether the same sample was measured repeatedly
☒	☐	The statistical test(s) used AND whether they are one- or two-sided<br>*Only common tests should be described solely by name; describe more complex techniques in the Methods section.*
☒	☐	A description of all covariates tested
☒	☐	A description of any assumptions or corrections, such as tests of normality and adjustment for multiple comparisons
☐	☒	A full description of the statistical parameters including central tendency (e.g. means) or other basic estimates (e.g. regression coefficient) AND variation (e.g. standard deviation) or associated estimates of uncertainty (e.g. confidence intervals)
☒	☐	For null hypothesis testing, the test statistic (e.g. *F*, *t*, *r*) with confidence intervals, effect sizes, degrees of freedom and *P* value noted<br>*Give P values as exact values whenever suitable.*
☐	☒	For Bayesian analysis, information on the choice of priors and Markov chain Monte Carlo settings
☒	☐	For hierarchical and complex designs, identification of the appropriate level for tests and full reporting of outcomes
☒	☐	Estimates of effect sizes (e.g. Cohen's *d*, Pearson's *r*), indicating how they were calculated

Our web collection on statistics for biologists contains articles on many of the points above.

Software and code

Policy information about availability of computer code

Data collection	Not applicable - no software used
Data analysis	BayesTraitsV3.0 (Multistate), R v4.0.2, (packages: ape, bayestestR, brms, geiger, rstan). Code and command files for all phylogenetic analyses are provided on the OSF (https://osf.io/cm53v/).

For manuscripts utilizing custom algorithms or software that are central to the research but not yet described in published literature, software must be made available to editors and reviewers. We strongly encourage code deposition in a community repository (e.g. GitHub). See the Nature Portfolio guidelines for submitting code & software for further information.

Data

Policy information about availability of data

All manuscripts must include a data availability statement. This statement should provide the following information, where applicable:

- Accession codes, unique identifiers, or web links for publicly available datasets
- A description of any restrictions on data availability
- For clinical datasets or third party data, please ensure that the statement adheres to our policy

Data are publicly available on Pulotu (pulotu.com), as well as the OSF (https://osf.io/cm53v/).

Field-specific reporting

Please select the one below that is the best fit for your research. If you are not sure, read the appropriate sections before making your selection.

☐ Life sciences ☒ Behavioural & social sciences ☐ Ecological, evolutionary & environmental sciences

For a reference copy of the document with all sections, see nature.com/documents/nr-reporting-summary-flat.pdf

Behavioural & social sciences study design

All studies must disclose on these points even when the disclosure is negative.

Study description	This is a cultural phylogenetic study that uses quantitative ethnographic data coded from qualitative sources such as ethnographies, and incorporates previous work on the phylogenetic relationships between the societies in the sample. The evolution of cultural traits is modelled under different assumptions.
Research sample	The societies in the sample are 97 Austronesian-speaking peoples represented in the ethnographic literature. This sample was chosen because Austronesian language relationships are well-understood, and Austronesian societies are diverse and well-documented.
Sampling strategy	Societies chosen based on the availability of ethnographic sources and information on their phylogenetic relationships. We took a pre-existing reconstruction of the Austronesian language family as our starting point. This reconstruction (by Gray, Drummond and Greenhill, 2009) included 400 languages from all major subgroups of the Austronesian language family. We included every language that could be linked to a society for which reliable ethnographic data existed. No power analyses were conducted, but given this sampling strategy it is unlikely that the sample could have been much enlarged.
Data collection	The dataset was created by consulting ethnographic materials and coding the variables of interest (initially in a word document, later in an Excel spreadsheet) using pre-specified criteria. The researcher was not blind to the study hypotheses.
Timing	A preliminary version of the dataset was coded in June 2017, but revisions continued to be made until May 2021 as the variables of interest evolved and more ethnographic materials were read.
Data exclusions	Languages that could not be matched to a society for which adequate ethnographic data were available were excluded. Languages that were spoken by one of the societies in the sample but did not have the largest number of speakers according to Ethnologue were also excluded, based on the requirement of only one language per society. Our final sample included 97 languages out of the 400 in Gray, Drummond and Greenhill (2009), meaning that 303 were 'excluded'.
Non-participation	No participants were involved in the study.
Randomization	We aimed to include all societies that could be matched to a language in the tree and for which detailed ethnographic data was available, making randomisation inapplicable.

Reporting for specific materials, systems and methods

We require information from authors about some types of materials, experimental systems and methods used in many studies. Here, indicate whether each material, system or method listed is relevant to your study. If you are not sure if a list item applies to your research, read the appropriate section before selecting a response.

Materials & experimental systems

n/a	Involved in the study	
☒	☐	Antibodies
☒	☐	Eukaryotic cell lines
☒	☐	Palaeontology and archaeology
☒	☐	Animals and other organisms
☒	☐	Human research participants
☒	☐	Clinical data
☒	☐	Dual use research of concern

Methods

n/a	Involved in the study	
☒	☐	ChIP-seq
☒	☐	Flow cytometry
☒	☐	MRI-based neuroimaging
